# How mutualisms arise in phytoplankton communities: building eco‐evolutionary principles for aquatic microbes

**DOI:** 10.1111/ele.12615

**Published:** 2016-06-10

**Authors:** Elena Kazamia, Katherine Emma Helliwell, Saul Purton, Alison Gail Smith

**Affiliations:** ^1^ Department of Plant SciencesUniversity of CambridgeDowning StreetCambridgeCB2 3EAUK; ^2^ Institute of Structural and Molecular BiologyUniversity College LondonGower StreetLondonWC1E 6BTUK; ^3^ Present address: The Marine Biological Association of the UKCitadel HillPlymouthPL1 2PBUK

**Keywords:** Eco‐evolutionary dynamics, Foraging‐to‐Farming hypothesis, metabolite exchange, metagenomics, microbial communities, mutualism, phytoplankton, vitamins

## Abstract

Extensive sampling and metagenomics analyses of plankton communities across all aquatic environments are beginning to provide insights into the ecology of microbial communities. In particular, the importance of metabolic exchanges that provide a foundation for ecological interactions between microorganisms has emerged as a key factor in forging such communities. Here we show how both studies of environmental samples and physiological experimentation in the laboratory with defined microbial co‐cultures are being used to decipher the metabolic and molecular underpinnings of such exchanges. In addition, we explain how metabolic modelling may be used to conduct investigations in reverse, deducing novel molecular exchanges from analysis of large‐scale data sets, which can identify persistently co‐occurring species. Finally, we consider how knowledge of microbial community ecology can be built into evolutionary theories tailored to these species’ unique lifestyles. We propose a novel model for the evolution of metabolic auxotrophy in microorganisms that arises as a result of symbiosis, termed the Foraging‐to‐Farming hypothesis. The model has testable predictions, fits several known examples of mutualism in the aquatic world, and sheds light on how interactions, which cement dependencies within communities of microorganisms, might be initiated.

## Introduction

**Figure 1:**
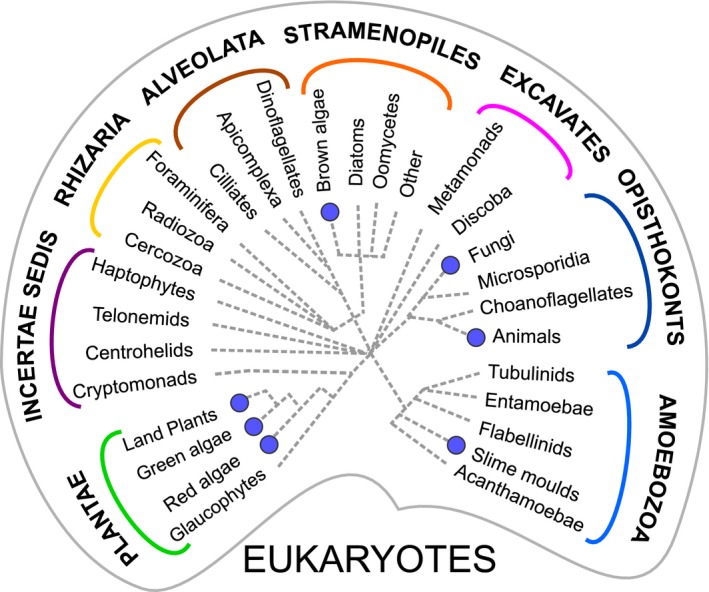
Unicellular organisms dominate the eukaryotic lineages. A schematic diagram of the eukaryotic tree of life showing the major groups (Dorrell & Smith (2011); Burki 2014). At present the positions of the Haptophytes, Telonemids, Cryptomonads and Centrohelids remain uncertain (*Incertae sedis*). Multicellularity has evolved only seven times (highlighted with filled circles); all other lineages are essentially microbial.

Microorganisms are the ‘unseen majority’ of life on Earth. As well as being numerically dominant, they also constitute the major phylogenetic diversity, even within the Eukaryotes where nearly every lineage is dominated by unicellular or microscopic species, and where multicellularity is the exception rather than the rule (Fig. 1). Moreover, in a range of ecosystems including the soil and ocean biomes, microorganisms make the major impact on global processes such as the biogeochemical cycling of carbon, nitrogen and sulphur (Falkowski *et al*. 2008; van der Heijden *et al*. 2008). They are particularly important in the aquatic environment because here a subset of species, the phytoplankton, which comprise both eukaryotic microalgae and cyanobacteria, are responsible for primary production, contributing an estimated 50% of the total global carbon fixation (Field *et al*. 1998), and sustaining all other trophic levels. However, despite their importance, our understanding of the ecology of the phytoplankton and the associated microorganisms in the photic zone is limited, due both to a lack of theoretical principles that are relevant to their unique lifestyles, as highlighted by Prosser *et al*. (2007), but also to the difficulty in studying many of these species in the laboratory. Aquatic microorganisms are notoriously difficult to isolate from the natural environment, as only an estimated 0.01–0.1% of oceanic marine bacterial cells produce colonies by standard diagnostic plating techniques (Connon & Giovannoni 2002), and most species lack morphological characters. One of the proposed explanations for the difficulty in isolating species is that community interactions, which are severed in axenic (single organism) laboratory cultures, are vital for the survival of microorganisms (e.g. Joint *et al*. 2010).

**Figure 2:**
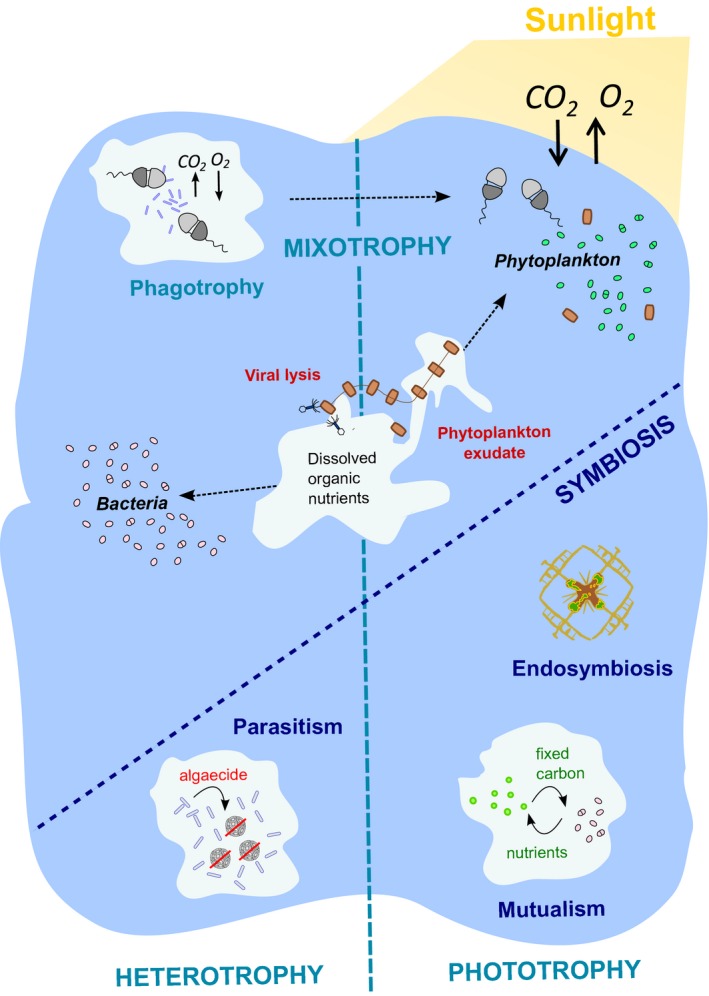
Schematic of phytoplankton lifestyles. Ecologically significant groups, including diatoms, bloom and are subject to viral lysis and grazing by heterotrophic zooplankton. This releases organic nutrients into solution, which both cyanobacteria and algae can utilise via mixotrophy. Moreover, many algae such as dinoflagellates consume bacterial prey via phagotrophy, resulting in net CO_2_ release and O_2_ consumption. In addition to these trophic processes is a complex series of interactions, or symbioses, which have shaped the ecology and evolution of microbes in aquatic communities. Many examples of mutualism are known, where algae supply fixed carbon (photosynthate) in exchange for specific nutrients such as vitamins. Parasitism can also arise, as in the case of senescing haptophytes, where bacterial partners that were initially mutualistic produce algaecides to accelerate the process, indicating that interactions can be dynamic. Intimate physical associations, in the form of endosymbiosis, such as between the amoeboid protistan radiolarians and haptophytes, are also frequent.

Photosynthetic algae are at the start of most aquatic food chains, and their consumption by zooplankton is the first link in a classic aquatic trophic cascade. However, many planktonic algae have more complex lifestyles (Fig. 2). Mixotrophy, where photosynthesis is combined with uptake of dissolved organic carbon, is common throughout the algal lineages. Alongside osmotrophic uptake of dissolved organic carbon (osmotrophy), representatives of several ecologically significant groups such as dinoflagellates, carry out phagotrophy by grazing on bacterioplankton prey (Jones 2000). Evidence from field studies in the North Atlantic ocean have demonstrated that photosynthetic mixotrophs can account for a staggering 40–95% of bacterivory in certain regions of the ocean (Hartmann *et al*. 2012). This complements laboratory‐based studies, which have demonstrated phagotrophy in eukaryotic phytoplankton species typically considered strict autotrophs, such as the picoalga *Micromonas (*McKie‐Krisberg & Sanders 2014)*,* a particularly remarkable example given its tiny cell size (< 2 μm). As such, mixotrophy is increasingly being recognised as a major contributor to plankton dynamics, challenging the classic distinction made between ‘phototrophic’ phytoplankton and heterotrophic zooplankton (Flynn *et al*. 2012).

Similarly, defining symbiotic interactions between aquatic microbes is equally complex, since these often defy strict categorisation (with only a subset of examples shown in Fig. 2). For example corals are one of the better‐studied aquatic associations, where a cnidarian exchanges metabolites with a dinoflagellate from the *Symbiodinium* genus. Traditionally, this has been considered a classic case of mutualism, as inorganic waste metabolites from the animal host are exchanged for organic nutrients fixed by dinoflagellate photosynthesis (Muscatine & Porter 1977), benefitting both partners. However, experimental evidence shows that there is considerable functional diversity within the *Symbiodinium* lineage, with certain clades interacting with hosts in a manner that is closer to parasitism, by fixing and releasing significantly less carbon than close relatives also capable of the association (Stat *et al*. 2008). Furthermore, some species of *Symbiodinium* have been shown to switch their behaviour from mutualism to parasitism depending on the manner of transmission. *S. microadriaticum,* which infects jellyfish as an endophotosymbiont, was experimentally enforced to infect its host through horizontal transmission (defined as infectious transfer among unrelated hosts) and this selected for an evolutionary shift to parasitism (Sachs & Wilcox 2006). Similarly, it has been observed that within the dynamic environment of open oceans, mutualisms underpinning algal blooms may turn to parasitism as the bloom reaches its climax and fades, often accompanied by sudden and extensive viral lysis (e.g. Yager *et al*. 2001).

That microbial interactions in the aquatic realm do not fit known ecological modes of interaction is not surprising, since historically these have been formulated to explain life in terrestrial biomes. Perhaps the simplest way of interpreting aquatic microbial interactions, on a fundamental level, is through the lens of characterising the metabolism of the constituent species. We propose that symbioses in this context should be classified as ‘active metabolic associations between two or more organisms, with an implied ecology’ to distinguish from the passive interactions that can be a by‐product of living in a shared environment. Passive interactions would include nutrient exchange through metabolic by‐product exudates, or following virus‐induced lysis of cells. These give rise to a dissolved pool of organic nutrients, made available to species either at a different depth or significantly later in time, in pace with circulation events such as seasonal upwelling along continental margins, in a process known as the ‘microbial loop’ (Azam *et al*. 1983). Similarly, trophic interactions would not be strictly symbiotic as they constitute an active behaviour on the part of only one of the species, rather than both. Following this definition, a symbiotic interaction would require that both species are alive during their association and affect each other's metabolism, cellular functions and lifestyle. While physical associations are possible and frequent in the microbial world, in our view they are not a pre‐requisite for symbiosis, as metabolic exchanges could happen without contact. We discuss the challenges associated with studying the physical aspects of microbial interactions later in the paper. Furthermore, we consider that while not all symbiotic interactions are specific, this should be a pre‐requisite for mutualism, which implies recurrent interactions and the potential for co‐evolution. Nonetheless, the mutualism need not be permanent, but might operate under particular environmental conditions, or stage of the life cycle.

An important development in recent years, which is revolutionising the study of microbial communities, is that of ‘omics methodologies that can collect whole systems data from environmental samples, including aquatic ecosystems. Sequencing of marker genes such as 16S rRNA genes for prokaryotes and 18S rRNA for eukaryotes has allowed the ‘metabarcoding’ of the species diversity within these samples (Mende *et al*. 2013; de Vargas *et al*. 2015). This is a culture‐free method that can track microbial species richness across global transects. Furthermore, the sequencing of whole genomes from sampled communities, a practice referred to as metagenomics, has opened the possibility for comparisons of metabolic functions across samples, and metatranscriptomics and metaproteomics can confirm the expression of the genes and infer presence of function, which gives a snapshot of the ecological state of a community, allowing for comparisons in time or under different conditions (e.g. Moran *et al*. 2013). On the other hand, these whole state studies do not in themselves shed light on the specific interactions of the microbes within, which is only possible through physiological experimentation using defined systems. Nonetheless, there are cross‐links between the two. Co‐occurrence within metabarcoding data sets can be indicators of active interactions. Similarly, it is possible to assess whether a model laboratory system is widespread by looking for functional genes that reflect it in metagenomic or metatransciptomic compilations.

In the following review, we synthesise recent knowledge from these two complementary fields to draw conclusions on what they can tell us about mutualisms in aquatic communities, with particular reference to microalgae. Furthermore, we demonstrate how mechanistic understanding of a specific interaction can explain eco‐evolutionary aspects, leading to establishment of theoretical principles in the burgeoning field of microbial ecology.

## Whole systems approaches to studying interactions within microbial communities

The advent of affordable high‐throughput genomic analyses has uncovered unexpected microbial diversity from a range of biomes, including soil and aquatic environments, and has expanded our horizons of the ‘known unknowns’ of all extant life on earth (e.g. Pace 1997). Once it was possible to catalogue the genetic diversity into data sets, this opened the door for investigations into the function of genes within organisms as well as between interacting species. Chaffron *et al*. (2010) presented one of the first studies of genomic correlates between diverse sampling sites. The purpose was to uncover consistent associations that could be indicative of ecological interactions. Using network clustering and co‐occurrence analyses the investigators were able to identify putative novel associations; for example, a lineage of cyanobacteria (belonging to the halophilic *Euhalothece*) was observed to be associated with an uncharacterised lineage having no cultivated or named representatives (a monophyletic sister group of the *Psychroflexus* lineage of bacteroidetes).

The Sorcerer Global Ocean Survey (GOS) was the first global scale effort to obtain metagenome DNA sequences from communities of marine microbes (Venter *et al*. 2004). It obtained 6.3 gigabases of DNA sequences from surface‐water samples collected along a transect from the Northwest Atlantic to the Eastern Tropical Pacific. Information from the GOS together with 30 smaller scale independent projects contributed towards the Census of Marine Life and the International Census of Marine Microbes (ICoMM). Analysis of the data compiled by ICoMM uncovered the ‘rare biosphere’ of low‐abundance microbial populations that account for most of the observed phylogenetic diversity in the deep ocean, and represent an inexhaustible source of genomic innovation (Sogin *et al*. 2006). Interrogation of microbial metagenomic sequence data collected as part of the Sorcerer II Global Ocean Expedition revealed a high abundance of viral sequences, representing approximately 3% of the total predicted proteins (Williamson *et al*. 2008). While the GOS projects clearly represent an important advance in allowing microbial species composition and diversity to be catalogued on global scales, species composition analysis was confined to prokaryotic organisms, and there was little consideration of interactions between the species.

The Tara Oceans Expedition, which was unveiled in the spring of 2015, superseded all previous efforts in its magnitude, collecting ~ 35 000 samples across multiple depths at a global scale over a period of 3 years (Bork *et al*. 2015; de Vargas *et al*. 2015). The extent of the data set has provided the means to determine the microbial ‘interactome’. For example Lima‐Mendez *et al*. (2015) focussed on data from viruses to small metazoans collected at 68 ocean stations. Co‐occurrence detection techniques were applied to data sets sub‐classified into kingdoms (e.g. data exploring eukaryote diversity focused on the hyper variable V9 region of the 18S rRNA genes only) and also across kingdoms. Machine learning techniques applied to the integrated network predicted 81 590 interactions based on correlations, and found that the majority (~ 78%) of these were positive, that is to say the presence of one species provided a supporting role for another. Largely, these reflected the nature of trophic cascades, for example zooplankton were commonly associated with their preferred food, and parasites were associated with their hosts, but interestingly, there were also a range of positive interactions observed between planktonic microorganisms belonging to the same size category. The basis for these interactions remains unknown, but it is possible that they represent mutualisms. Mining of the Tara interactome confirmed that previously known mutualisms were captured by the network analysis, such as the symbiosis between diatoms and flavobacteria (Jolley & Jones 1977), and dinoflagellate associations with members of Rhodobacterales (*Ruegeria* sp.) (Miller & Belas 2004). Moreover, certain interactions predicted by the study could then be confirmed experimentally. For example an endosymbiotic photosymbiotic interaction between an acoel flatworm (*Symsagittifera* sp.) and a green microalga (*Tetraselmis* sp.) was predicted by consistent V9‐V9 co‐occurrence in the Tara data set. Fifteen acoels specimens collected independently were then used to validate the interaction, both by laser scanning confocal microscopy and by molecular biology through single cell 18S rDNA sequencing.

One of the main limitations of metagenomics and metabarcoding, however, is that it does not link metabolic capabilities directly to a particular species within the community. At best, analysis of sequence data provides correlations, and confirmation of direct interactions requires physiological and biochemical data, which are challenging to obtain with environmental samples. Metatranscriptomics and metaproteomics can arguably provide a bridge to link the two. Such analyses can demonstrate the executed functions within microbial communities at time of sampling, and often reveal unexpected information. For example while metagenomic analysis of biofilms on the hulls of naval vessels indicated a preponderance of bacteria, the majority of proteins identified by metaproteomics using liquid chromatography‐tandem mass spectrometry were eukaryotic (Leary *et al*. 2014). Quantitative ^18^O and iTRAQ analyses, coupled with measurement of photosynthetic pigments confirmed that the communities were dominated by diatoms. Similar inferences about the functional dynamics of aquatic communities can be made from metatranscriptomic analyses, and have been used to good effect to monitor successions during algal blooms, (Cooper *et al*. 2014), to establish the effect on the bacterial population associated with the bloom (Wemheuer *et al*. 2014), or to show how nutrients affect niche differentiation (Harke *et al*. 2016).

## Studying the metabolic basis for mutualisms between microbes

Despite the considerable information that can be gained from studies of environmental samples, inferring and characterising interactions is at best correlative – a process once described as ‘akin to boiling dinner leftovers in a pot for 24 h, pureeing heavily and then trying to attribute any spice or stew fragment back to the original dish or constituent from which it derived’ (Heidelberg *et al*. 2010). To gain real mechanistic understanding of interactions it is necessary to study defined or model systems that can be co‐cultured under controlled conditions, enabling identification of the metabolic foundations for the ecology, demonstrating directly the compounds that are exchanged, and studying the molecular machinery involved in the associations. Importantly, studies of model systems generate testable hypotheses of how the interactions might have evolved, which in turn will allow development of eco‐evolutionary principles.

The lifestyle of a unicellular organism is constrained by its individual metabolism. Metabolic requirements that have to be fulfilled to ensure survival but cannot be satisfied by the abiotic environment create the ecological niche for mutualism. For example microorganisms vary in their capacity to access essential elements from inorganic molecules, frequently requiring them in a specific ‘bioavailable’ form that is dependent on the metabolic activity of other species.

Historically, the study of metabolic requirements in aquatic microorganisms has focused largely on the adaptations of individual species for acquiring nutrients, the latter classified as either macro (C, N, O, H, P and S) or micro depending on the quantity required. Micronutrients are required in much lower amounts, but have essential functions. These include the mineral micronutrients (e.g. Ni, Mo, Zn, Cu, Mn, Fe and Co), involved in protein function, enzyme catalysis and electron transfer reactions, and a range of organic vitamins required for enzyme cofactors. A particularly important example is iron, which is limiting to algal growth in much of the world's oceans, including the equatorial Pacific and Southern Oceans (Behrenfeld *et al*. 1996; Behrenfeld & Kolber 1999). Many bacteria produce siderophores, organic chelators of both Fe^II^ and Fe^III^, which allow them to sequester this scarce nutrient. Specific mutualistic interactions have been described in which microalgae engage with heterotrophic bacteria to access this source of chelated Fe. For example several clades of *Marinobacter* found in close association with two major groups of marine algae, dinoflagellates and coccolithophores, produced an unusual siderophore, vibrioferrin (VF), which when chelated to iron undergoes photolysis at rates that are 10–20 times higher than siderophores produced by free‐living marine bacteria (Amin *et al*. 2009). Experiments confirmed that photolysis of the chelates increased algal uptake of Fe‐VF by > 20‐fold.

It is widely acknowledged that the two macronutrients most limiting to phytoplankton are nitrogen and phosphorus (Tyrrell 1999). Only certain prokaryotic species (‘diazotrophs’) are capable of fixing the inert dinitrogen gas into bioavailable forms. In the photic zones of the ocean, these diazotrophs are mainly cyanobacterial species of various morphologies and lifestyles (Zehr & Kudela 2011). A proportion of these exchange fixed nitrogen for photosynthate with diatoms, in a symbiosis referred to as diatom‐diazotroph associations. For example *Richelia intracellularis* and *Calothrix rhizosoleniae*, filamentous heterocyst‐forming cyanobacteria, have been found in mutualism with several diatom genera, including *Hemiaulus*,* Rhizosolenia* and *Chaetoceros* (Zehr & Ward 2002). The interactions are highly dynamic, demonstrated by the fact that the length, location and number of *Richelia* and *Calothrix* trichomes per diatom partner, and phylogeny of the symbionts, differ in each symbiosis (Jahson *et al*. 1995; Foster & Zehr 2006).

Unicellular nitrogen‐fixing cyanobacteria also are thought to interact with microalgae. Using metagenomic techniques, Zehr *et al*. (2003) identified two novel groups of unicellular diazotrophic picocyanobacteria, UCYN‐A and UCYN‐B (now formally classified as *Crocosphaera watsonii*). Whole‐genome amplification of UCYN‐A (Candidatus *Atelocyanobacterium thalassa*) revealed that it lacks genes for photosystem II and the Krebs and Calvin cycles, but retains sufficient electron transfer capacity through alternative electron donors to generate energy and reducing power from light (Tripp *et al*. 2010). Since this metabolic capability is insufficient for an autotrophic lifestyle, it was hypothesised that UCYN‐A was mutualistic with photosynthetic picoeukaryotes (PPEs), with which it frequently co‐occurred. Microscopy of individual cells coupled to secondary ion mass spectrometry (referred to as nano‐SIMS), in combinations with halogen *in situ* hybridisation confirmed the interaction visually by demonstrating the delivery of carbon from the PPEs to UCYN‐A cells (Thompson *et al*. 2012). This is a powerful example of how single cell imaging techniques can be used to show directly the transfer of metabolites between interacting species. Further investigations revealed that at least two different UCYN‐A phylotypes exist (Bombar *et al*. 2014), the clade UCYN‐A1, which is predominantly coastal and found in symbiosis with an uncultured small prymnesiophyte, and the clade UCYN‐A2, its open ocean relative which is found in symbiosis with the larger *Braarudosphaera bigelowii*. Studying the geographical distribution of UCYN‐A1 and UCYN‐A2 and symbionts recorded within the Tara data set revealed a strikingly consistent co‐occurrence (Cabello *et al*. 2015), despite the absence of conclusive evidence for physical association in these relationships.

Mutualism based on vitamin exchange has also been demonstrated between microalgae and heterotrophic bacteria (e.g. Croft *et al*. 2005; Wagner‐Döbler *et al*. 2010). Over half of microalgal species surveyed across all lineages require cobalamin (vitamin B_12_), ~ 22% require thiamine (B_1_) and 5% require biotin (B_7_) (Croft *et al*. 2006). In the case of thiamine and biotin, auxotrophic species have lost the ability to synthesise the metabolite *de novo*, although often retain parts of the metabolic pathway. For example thiamine‐requiring algae are commonly able to survive on one or more biosynthetic intermediates (McRose *et al*. 2014). This is also true for heterotrophic bacteria that are B‐vitamin auxotrophs. Representatives belonging to the abundant and ubiquitous SAR11 clade of marine chemoheterotrophic bacteria have genes for all the thiamine biosynthetic enzymes except for *thiC*, encoding an enzyme required for the synthesis of the pyrimidine moiety, 4‐amino‐5‐hydroxymethyl‐2‐methylpyrimidine (HMP) (Carini *et al*. 2014). Interestingly, the authors found that while the SAR11 isolate ‘Candidatus *Pelagibacter ubique*’ was able to grow on HMP, addition of thiamine itself to laboratory cultures did not rescue growth. The authors attribute this to the absence of thiamine transporters in the Ca. *P. ubique* genome. They propose that vitamin cycling mediated through partial precursors and the presence/absence of transporters requires cooperation and interactions within marine microbial communities. Another study by Paerl *et al*. (2015) investigated thiamine cycling between the marine bacterium *Pseudoalteromonas* sp. TW7 and cosmopolitan marine picoalga *Ostreococcus lucimarinus* CCE9901. The bacterium was able to enhance the bioavailability of the pyrophosphorylated form of the vitamin, thiamine pyrophosphate (TPP) to *O. lucimarinus*. Bacterial phosphatase activity was inferred to metabolise TPP to thiamine monophosphate, which is better able to support *O. lucimarinus* CCE9901.

Non‐requirers of thiamine and biotin are able to synthesise the compounds for their own metabolism but the underlying genetic reason for vitamin B_12_ auxotrophy in algae is quite different. This compound is made only by prokaryotes (Warren *et al*. 2002), in a process that requires more than 20 enzyme‐catalysed steps from the common tetrapyrrole precursor. Despite the widespread occurrence of B_12_ requirement amongst algae, there is no phylogenetic relationship between them, with auxotrophs and non‐auxotrophs often found within the same genus, for example *Chlamydomonas nivalis* requires B_12_, but *C*. *reinhardtii*, does not. Vitamin B_12_ is used primarily as an essential cofactor for the enzyme methionine synthase (METH), the key enzyme of one‐carbon (C1) metabolism. An alternative B_12_‐independent methionine synthase (METE) exists, but is catalytically less efficient than METH (Gonzalez *et al*. 1992). Auxotrophs have METH only, whereas non‐requirers either have both enzymes, as is the case for *C. reinhardtii*, or METE only, such as *Coccomyxa* sp. C‐169 and *Cyanidioschyzon merolae* (Croft *et al*. 2005; Helliwell *et al*. 2011), suggesting that B_12_‐dependence arose through the loss of *METE* numerous times in algal evolution. Helliwell *et al*. (2015) were able to demonstrate this experimentally in *C. reinhardtii,* which lost a functional copy of *METE* in fewer than 500 generations of vitamin B_12_ supplementation, indicating of the ease with which this might have occurred in other lineages.

The most comprehensive analysis of vitamin B_12_ concentrations in the ocean was along a transect off the coast of California (USA), which detected little to no vitamin B_12_ in surface ocean waters (Sañudo‐Wilhelmy *et al*. 2012), providing evidence that this organic micronutrient might be limiting for algal requirers. Indeed enrichment experiments showed that addition of B_12_ stimulates phytoplankton growth in samples of water collected from temperate coastal areas (Sañudo‐Wilhelmy *et al*. 2006; Gobler *et al*. 2007), the Southern Ocean's Gerlache Straight (Panzeca *et al*. 2006), and the Ross Sea (Bertrand *et al*. 2007). Since only bacteria are capable of B_12_ biosynthesis, they must be the ultimate source of the vitamin, and it has been suggested that acquisition is through mutualism with bacterial producers (Croft *et al*. 2005). Several laboratory co‐cultures have been described where there is delivery of vitamin B_12_ from bacteria to algae in exchange for photosynthate, including for members of the Chlorophyta (Croft *et al*. 2005; Kazamia *et al*. 2012), Alveolata (Wagner‐Döbler *et al*. 2010), and diatom (Heterokontophyta) lineages (Durham *et al*. 2014). Even though many of the co‐cultures were the result of artificial combination, they are often exceptionally stable. For example co‐cultures of the green alga *Lobomonas rostrata* with the alpha‐proteobacterium *Mesorhizobium loti* persist at the same algal : bacterial ratio (of ~ 1 : 30) over many rounds of sub‐culturing (Kazamia *et al*. 2012), and follow predictable dynamics indicative of regulation (Grant *et al*. 2014). Interestingly, not all combinations of bacterial producers and algal auxotrophs led to stable co‐cultures (Kazamia *et al*. 2012), which is indicative of species‐specific associations and not simple metabolic fitting.

Looking for evidence of symbioses between algae and bacteria for vitamin B_12_ acquisition in the natural environment, Bertrand *et al*. (2015) identified *Oceanospirillaceae* ASP10‐02a as a possible vitamin B_12_ producer in sea‐ice edge microbial communities from the Southern Ocean, which are characterised by extensive blooms of diatoms during the Antarctic summer. Metatranscriptomics data showed that *Oceanospirillaceae* ASP10‐02a contributed more than 70% of reads from cobalamin biosynthesis‐associated genes, and peptides of one particular enzyme, CbiA, from this species were abundant in a proteomics data set. Interestingly, they also observed transcripts for proteins involved in uptake and salvage of B_12_ from other bacteria, such as *Methylophaga,* which does not encode the complete biosynthetic pathway. This implies several different phytoplankton–bacterial interactions for provision of this organic micronutrient that might involve both positive and negative feedback loops.

**Figure 3:**
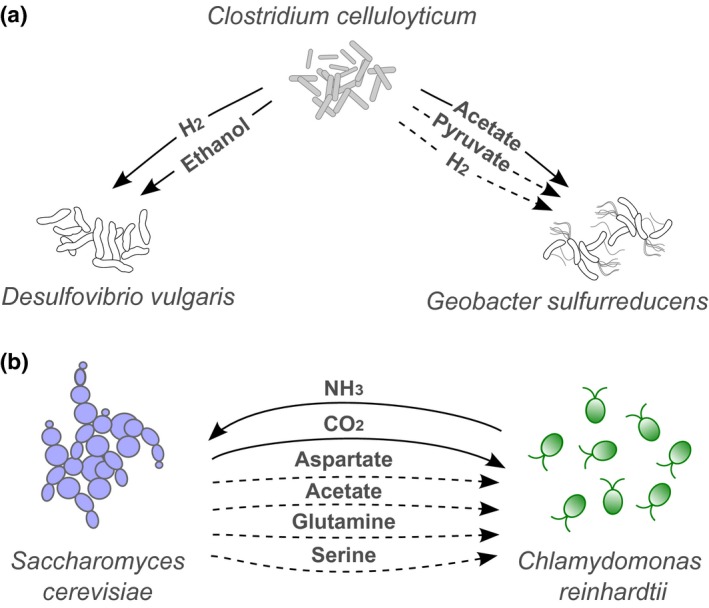
Microbial systems with well‐characterised metabolic exchanges. These have been used to validate the SMETANA algorithm devised by Zelezniak *et al*. (2015). In each case solid arrows represent known exchanges tested in physiological studies and dotted arrows mark potential novel interactions predicted by SMETANA. (a) A three‐species community based on cellobiose degradation (Miller *et al*. 2010). SMETANA predicts that pyruvate and hydrogen may also be delivered to *G. sulfurreducens* by *C. cellulolyticum*. (b) An algal‐fungal mutualism between *C. reinhardtii* and *Saccharomyces cerevisiae* (Hom & Murray 2014). As well as refining the likely forms of N and S that are exchanged, SMETANA predicted that during co‐culture growth aspartate, glutamine and serine could also be delivered from the yeast to the alga.

The above examples describe studies of microbial interactions where the metabolic requirement or auxotrophy is known. However, is it possible to infer metabolic exchanges based on known recurring species correlations? The large data sets cataloguing microbial diversity offer this possibility. Zelezniak *et al*. (2015) analysed a compilation of 16S rRNA sequences to obtain the species composition for 1297 communities from habitats including soil, water and the human gut. For 261 species whose genomes are sequenced and mapped, the authors constructed whole‐genome metabolic models using the ModelSEED pipeline (Henry *et al*. 2010). Two metrics, the metabolic resource overlap (MRO) and the metabolic interaction potential (MIP), were used as indicators for competition or possible mutualism respectively. On average, communities had higher MRO scores than predicted by chance, and intriguingly the authors found high MIP scores in nutritionally rich habitats where incentive for metabolic cross‐feeding would be predicted to be lower. The ‘Species METabolic interaction ANAlysis’ (SMETANA) algorithm was used to identify metabolic exchanges in a community that was modelled as living on minimal medium. It predicted that the most frequently exchanged metabolites were amino acids and sugars. SMETANA was verified against a well‐studied three‐species bacterial community (Fig. 3a; Miller *et al*. 2010), and co‐cultures of the yeast *Saccharomyces cerevisiae* and *C. reinhardtii* (Hom & Murray 2014), summarised in Fig. 3b. The model correctly reproduced known exchanges and identified further possible links in the system, namely that *S. cerevisiae* might also be able to deliver aspartate, glutamate, glutamine and serine to *C. reinhardtii,* although there is as yet no experimental evidence that *C. reinhardtii* can be supported by these nutrients. This highlights an important limitation of bioinformatics approaches when used in isolation, namely that genetic capacity may not accurately predict physiology. A further example of this comes from studies of the production and use of vitamin B_12_ amongst different phytoplankton classes. Cyanobacteria are known producers of B_12_, and have been proposed to be a major source of the vitamin to B_12_‐requiring eukaryotic algae (Bonnet *et al*. 2010). However, biochemical and physiological investigations revealed that cyanobacterial species such as strains of the abundant marine genus *Synechococcus* produce a form of B_12_, pseudocobalamin, which is considerably less bioavailable to eukaryotic algae (Helliwell *et al*. 2016).

## From metabolic fitting to complex regulatory dynamics

While exchange of nutrients is considered to be at the heart of associations between marine microorganisms, there is often more complicated ecology involved. For example Seyedsayamdost *et al*. (2011) describe a ‘Jekyll‐and‐Hyde’ association between the bloom‐forming coccolithophore *Emiliania huxleyi* and the roseobacter *Phaeobacter inhibens* (BS107). During a bloom, *E. huxleyi* can account for 80–90% of the phytoplankton in an area, and up to 60% of the bacterial community belong to the roseobacter clade (Alpha‐proteobacteria) (González *et al*. 2000), so associations between the two are thought likely. The reduced system described by Seyedsayamdost *et al*. (2011) was brought into the laboratory for analysis and exhibited two stages. Initially *P. inhibens* (BS107) promoted algal growth by biosynthesising auxins and antibiotics against algal competitors, and *E. huxleyi* released dimethylsulphopropionate (DMSP) in return, which the bacteria used as a carbon source. However, when the algae began to senesce, *P. inhibens* (BS107) switched its metabolism to the production and secretion of selective algaecides, killing their symbionts and switching to parasitism (Fig. 2).

A further hormone‐regulated interaction between algae and bacteria was described by Amin *et al*. (2015), who showed that cultures of the ubiquitous diatom *Pseudo‐nitzschia multiseries* were regulated by a *Sulfitobacter‐*related species of bacterium, referred to as SA11, through the secretion of the auxin indole‐3‐acetic acid. The association was chosen based on the previous observation of regular associations between different geographical isolates of *Pseudo‐nitzschia multiseries* originating from the Atlantic Ocean and the north Pacific Ocean with clades of bacteria belonging to Alpha‐proteobacteria (*Sulfitobacter*), Gamma‐proteobacteria (*Marinobacter*), Beta‐proteobacteria (*Limnobacter*) and Bacteroidetes (*Croceibacter*) (Amin *et al*. 2012). In the laboratory study, the *Sulfitobacter* cells were demonstrated to receive organic carbon required for growth, as well as taurine, a sulphonated metabolite from the diatom, and responded to stimulation by DMSP, which diatoms also produce. In return, the bacteria enriched the co‐culture medium with ammonium, the preferred nitrogen source for diatoms, by switching their own metabolic preference to nitrate (Amin *et al*. 2015).

The *Pseudo‐nitzschia* genus of diatoms includes bloom‐forming toxin‐producing species, as well as non‐toxic representatives. Working with isolates of *Pseudo‐nitzschia* collected from the Santa Cruz Wharf in California, Sison‐Mangus *et al*. (2014) compared the bacterial associations of toxic *Pseudo‐nitzschia* with non‐toxic species. Different diatom strains had their own unique bacterial communities. To investigate the physiology of interactions, transplant experiments were performed, where bacteria associated with one host were co‐cultured with a different *Pseudo‐nitzschia* isolate. A change in behaviour was shown for certain bacteria that were mutualistic to their native diatom but were commensal or parasitic to foreign hosts. Moreover, the algae exhibited plasticity with regards to domoic acid toxin production, depending on the bacterial species with which they were co‐cultured: less domoic acid was produced when in association with their cognate bacteria.

Further evidence of complex regulatory symbiotic interactions between marine microorganisms comes from the work of Decelle *et al*. (2012). Using molecular techniques in culture‐free studies it was shown that heterotrophic amoeboid protists belonging to the Acantharia (Radiolaria), which are some of the most abundant grazers in nutrient poor oceans, often engage in photosymbiosis with *Phaeocystis* species, a lineage of haptophyte eukaryotic microalgae ubiquitous in the marine environment. Photosymbioses between heterotrophic hosts and photosynthetic microalgae are widespread and prevalent in the oceanic plankton (Decelle *et al*. 2015). In the model interaction described by Decelle *et al*. (2012), more than 100 individual acantharian protists were isolated from seven geographical locations, and in each case were found to contain *Phaeocystis* cells in endosymbiosis with the host. There was no consistent relationship between the phylogenies of the interacting organisms, implying a facultative mutualism on the part of the *Phaeocystis*. Taken together with the observation that in each case the *Phaeocystis* species were also known to have an extensive free‐living population, the authors inferred that the symbiosis was selected for by local biogeography. However, the morphology of the haptophytes during the endosymbiosis was altered significantly, which suggests intricate metabolic associations that profoundly affect cellular structure and function and remain to be unravelled.

## Understanding how symbiosis affects evolution of microorganisms and *vice‐versa*

Within the natural environment, the complexity of microbial communities remains challenging to interpret both functionally (i.e. ecologically) and from an evolutionary perspective, particularly in the absence of theoretical models that link microbial ecology to evolutionary theory. A pivotal question about the evolution of interacting microorganisms is whether metabolic exchanges are the outcomes of stable ecological associations, or coincidental by‐products of these exchanges constrained by abiotic pressures.

In the oligotrophic oceans, habitats characterised by severe nutrient limitation, there is a strong selective pressure for streamlined genomes, leading to cells with lower DNA and associated protein content, and therefore lower cellular requirements for P and N (Dufresne *et al*. 2008). The dominant producers in the tropical oligotrophic oceans are picocyanobacteria belonging to the genus *Prochlorococcus* (Olson *et al*. 1990), which have the smallest genomes of any free‐living phototroph, with some isolates encoding only ~ 1700 genes (Rocap *et al*. 2003). Similarly, the dominant heterotrophs in these regions belong to the SAR11 clade. Ca. *P. ubique*, the first cultured member of the SAR11 clade, has ~ 1400 genes (Giovannoni *et al*. 2005). Although it encodes complete biosynthetic pathways for all 20 amino acids it has no pseudogenes, introns, transposons or extrachromosomal elements, and the shortest intergenic spacers yet observed for any cell.

However, reductive genome evolution often causes loss of key functions. For example *Prochlorococcus* has a smaller suite of oxidative‐stress genes than its closest relatives from the *Synechococcus* genus (Regelsberger *et al*. 2002). Specifically, no *Prochlorococcus* isolate encodes catalase‐peroxidase (*katG*), a haem‐dependent enzyme that is thought to be the primary defence against external hydrogen peroxide (H_2_O_2_) (Morris *et al*. 2008; Scanlan *et al*. 2009). Morris *et al* (2011) estimated that loss of this enzyme would reduce the cell quota for Fe in *Prochlorococcus* MED4 by 0.2%. Laboratory experiments demonstrated that *Prochlorococcus* grows better in the presence of heterotrophic bacteria, which reduce the concentrations of H_2_O_2_ (Morris *et al*. 2008). It was shown that these ‘helpers’ of *Prochlorococcus* such as *Alteromonas* EZ55 encode *katG,* and were able to deplete H_2_O_2_ from seawater to levels that were no longer toxic to *Prochlorococcus* (Morris *et al*. 2011).

**Figure 4:**
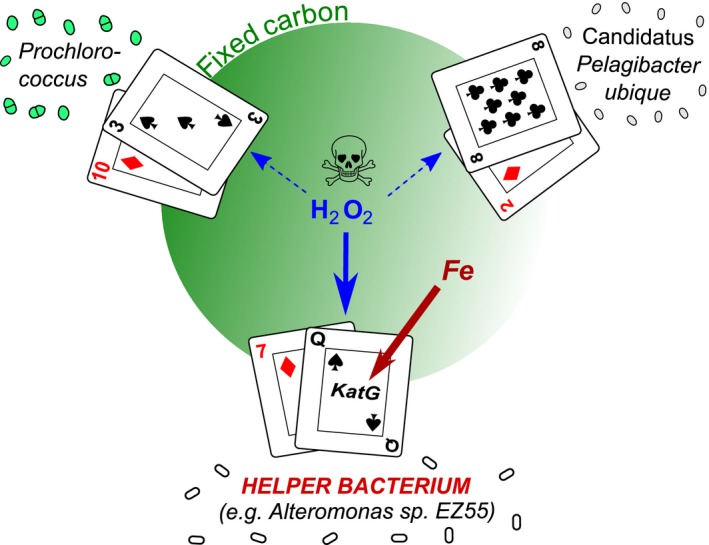
Black Queen Hypothesis (BQH). The Black Queen refers to the Queen of Spades in the card game Hearts, where players try to avoid ending up with this card, since it carries the greatest number of negative points. In the microbial community illustrated, the ability to detoxify H_2_O_2_ is analogous to the Queen of Spades, because it requires *katG,* an enzyme with a high Fe cost. Helper bacteria, such as *Alteromonas* sp. act as a sink for H_2_O_2_ and keep concentrations low enough for *Prochlorococcus* and other members of the aquatic community, including the numerically dominant heterotrophic Candidatus *Pelagibacter ubique*, to survive. *Prochlorococcus* is the photosynthetic producer in the system, fixing carbon that is made available to the other species in the community.

The work on *Prochlorococcus* led to the proposal of the Black Queen Hypothesis (BQH; Morris *et al*. 2012), a game‐theory model for the evolution of mutualism in microbial communities. To our knowledge, it is currently the only theoretical framework that links community microbial ecology to the evolution of dependency in individual species. The BQH (summarised in Fig. 4) posits that communities evolve to sustain a division of labour amongst the individual players. If an essential function is lost from a subset of species, this provides selection pressure for maintaining the provision of a shared ‘public good’ in helper species. In the case described, *Prochlorococcus* does not produce catalase but is protected from H_2_O_2_ by mutualist heterotrophs.

The BQH therefore applies only to ‘leaky’ functions and scenarios, where ‘public goods’ are traded against a background of severe nutrient limitation. This is in contrast to the prediction by Zelezniak *et al*. (2015) that interactions abound even under conditions of nutrient sufficiency. Moreover, experimental evolution approaches provide evidence that loss of function is the result of nutrient *sufficiency*. Growth of *C. reinhardtii* in elevated CO_2_ over 1000 generations resulted in several lines that exhibited a markedly reduced growth rate at ambient levels, (Collins & Bell 2004). Similarly, after ~ 500 generations in 1 μg L^−1^ B_12_ (Helliwell *et al*. 2015), a level > 100 higher than ambient (Sañudo‐Wilhelmy *et al*. 2012) a B_12_‐dependent *C. reinhardtii* mutant arose due to the loss of *METE* gene. It is worth mentioning that the evolved B_12_‐dependent clone of *C. reinhardtii* could be grown in co‐culture with B_12_‐synthesising rhizobial bacteria in medium without either B_12_ or a fixed carbon source, in apparently regulated mutualism.

**Figure 5:**
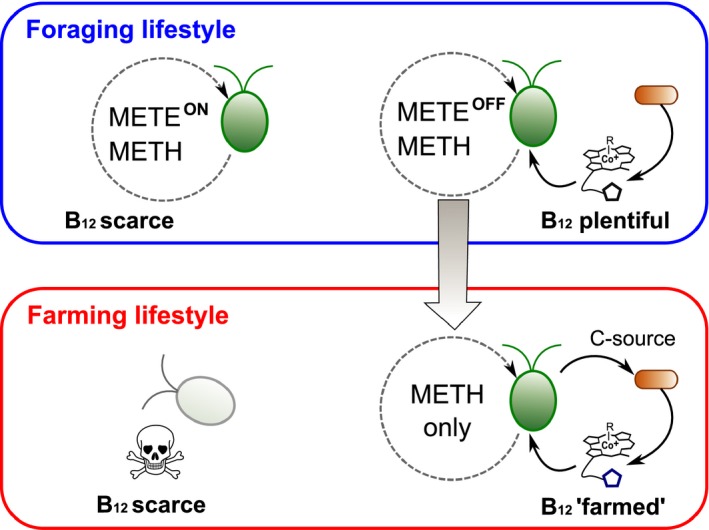
Evolution of B12 auxotrophy in algae, an example of the Foraging‐to‐Farming hypothesis. Algae with both isoforms of methionine synthase (METE and METH) are unhindered in the absence of B12 (Helliwell et al. 2011), but remain facultative users of the vitamin if available, much like foragers that take advantage of sporadic resources in their environment. If a persistent supply of the vitamin is available for sufficient time, for example from surrounding loosely associated bacteria, the METE gene will be repressed and may be lost, so that the alga is now completely dependent on bacteria for survival. However, fluctuating environmental conditions may mean that vitamin B12 becomes scarce. In such circumstances algae that release a carbon source will actively maintain a viable population of bacteria, and will consequently have a selective advantage over those that do not. Here, ‘farming’ the bacteria for the resource becomes an evolutionarily stable strategy, turning a previously loose interaction into an obligate one.

Taking these observations further it is possible to propose a model for the evolution of mutualism among algal lineages generally, using the delivery of B_12_ as example. If a B_12_‐independent alga such as *C. reinhardtii* that can use the vitamin when available (a ‘Forager’) is in contact for prolonged periods with a bacterium that synthesises B_12_, this might cause the loss of the algal *METE*. At this point the alga will be dependent on the bacterial B_12_‐producer, and it may well then change its behaviour to ‘farm’ the bacterium, providing photosynthate or other nutrients, to ensure its persistence within the vicinity, and therefore a secure supply of an essential nutrient (Fig. 5). This hypothesis, which we term *Foraging‐to‐Farming,* is an alternative but complementary scenario to the BQH. The Foraging‐to‐Farming model proposes that mutualism can evolve as accidental consequence of metabolic exchanges, under fluctuating conditions of resource availability. The starting point assumes the ability for both dependent and independent lifestyles, but dependency evolves as a consequence of recurrent ecological associations, which loosen the pressure to maintain the genetic capacity required for independence, thus reinforcing the mutualism. In the evolved *metE* mutant of *C. reinhardtii* (Helliwell *et al*. 2015) dependence is a consequence of a transposable element inserting into a single gene, disrupting its function, but it is possible to envisage multiple pathways to genetic degradation. Once genes are no longer essential, the lack of selection pressure means that they can accumulate deleterious mutations due to drift. Evidence for this comes from the presence of *METE* pseudogenes in several B_12_‐dependent algal species (Helliwell *et al*. 2011).

Such proposed origins for the evolution of dependency are not confined to B_12_, but have been found for other vitamins (Helliwell *et al*. 2013). For instance analysis of the genomes of individual members of the symbiotic microbiome of the tsetse fly gut has revealed inactivation of genes involved in thiamine biosynthesis, suggesting symbionts may ‘divide the labour’ of producing this compound, each contributing intermediates of different branches of the thiamine pathway, and thus sharing the cost of maintaining a supply of the vitamin (Belda *et al*. 2010). Thiamine biosynthesis genes in bacteria are subject to repression by elevated external levels of thiamine (Winkler *et al*. 2002), again providing a possible explanation for ease of their loss. Identification of pseudogenes of thiamine biosynthetic genes in both the eukaryote host and bacterial endosymbiont *Sodalis glossinidius* suggests this metabolic complementation may have occurred recently. Similarly, we propose our theorem could be applied to production of other metabolites, such as amino acids and nucleotides. In an innovative study, Campbell *et al*. (2015) devised a system using stochastic episomal segregation progressively to introduce metabolic auxotrophies into a population of *S. cerevisiae*. They found that despite a gradual loss of prototrophy for the metabolites histidine, leucine, uracil and methionine, self‐establishing communities of *S. cerevisiae* could maintain metabolic efficiency through cooperative metabolite exchange (Campbell *et al*. 2015). The observation that cells stopped making these metabolites in the presence of a ready‐available source from neighbouring cells could have contributed to the evolution of dependence, thus reinforcing a stable and obligate association, as would be predicted by the hypothesis.

The similarity between the BQH and the Foraging‐to‐Farming hypothesis is that they both lead to accidental dependency, as an ecological cul‐de‐sac. However, in the BQH this is driven by an abiotic pressure for streamlined genomes that leads to loss of function. For the Foraging‐to‐Farming model, the evolution of mutualism is driven by the presence of symbionts. There is an argument that the evolution of dependency is in the interest of the organisms being ‘farmed’, i.e. the providers of the public good or service. In the example we use, the farming lifestyle of the alga would guarantee a supply of fixed carbon for the vitamin B_12_ producers, and is therefore in the interest of the bacteria. The function does not have to be ‘leaky’, therefore, but could be selected for. The metabolic burden of producing B_12_ could be outweighed by the quantities of fixed carbon provided, a premise that can be tested experimentally. Examples of rapid symbiont‐driven evolution abound in insect‐endosymbiont literature (Himler *et al*. 2011; White 2011) but have never been reported for aquatic microorganisms. In contrast, the BQH assumes that the helpers are left burdened by the public function they deliver, essentially having not evolved fast enough to lose it, as other members of the community have. Being the last carriers of this function, loss of helpers from the community would lead to the collapse of the whole system. It is logical to hypothesise from this that their numbers in the community would therefore be as low as possible, without the total collapse of the system, another testable aspect of the model. In both instances, the net outcome is the partitioning of functions between members of stable communities, where the community metabolome, or ‘meta‐metabolome’, is streamlined to reduce redundancies.

## Placing evolutionary theories into an ecological context that is relevant for microorganisms

Valid evolutionary theories for microbial communities must be relevant for the physical environment that the microbes inhabit, while accommodating their unique lifestyles. In the absence of complex behaviour (as contrasted with macroorganisms), and a reliance on metabolic function as the main determinant of lifestyles, the boundary between ecology and evolution is blurred. The classical tenet that stable, repeated ecological associations lead to evolutionary outcomes simply may not hold, as evolutionary dynamics, manifested in genetic differences that result in metabolic dependencies, may precede and drive ecological associations. The fast growth rates of the species in question argue further in favour of blurring of the conventional distinction and directionality between ecology and evolution.

Moreover, it remains an open question whether physical associations are a pre‐requisite for stable associations and symbiosis, a tenet of classical terrestrial ecology (Boucher 1985). The work of Decelle *et al*. (2012) discussed above presents evidence of an ancient symbiosis, dated to approximately 92.8 Mya that requires contact but is nonetheless facultative, with species that are known to engage in endosymbiosis also abundant as free‐living organisms. It has also been argued that metabolic exchange could not be species‐specific, since this would require recognition of partners at a distance, which is not feasible in the marine environment (Droop 2007). However, developments in physical studies of the marine environment are redefining the concept of an ‘operational scale’ that is relevant to lifestyles of microbes (Stocker 2012). The argument in favour of directed interactions is that marine microorganisms are capable of chemotaxis, sensing and swimming not only along gradients of dissolved micronutrients but also towards other species (Grossart *et al*. 2001; Fenchel & Finlay 2004; Gärdes *et al*. 2011), with mean velocities exceeding 60–80 μm s^−1^ (Hütz *et al*. 2011); for comparison swimming speeds for *Escherichia coli* have been estimated at 15–30 μm s^−1^ (Chattopadhyay *et al*. 2006). At these speeds, the experienced distance between microorganisms is reduced, and species‐specific interactions may be possible. Moreover, the discovery of new physical microstructures in the ocean may play a role in bringing microbes into closer proximity. The role of ‘marine snow’ in creating microhabitats is long recognised (Alldredge & Silver 1988; Kiørboe & Jackson 2001), whereas other structures, such as marine microgels, have been characterised only recently. In the latter case, the bacterial abundance in local patches exceeds the seawater average by 10^4^ fold (Verdugo 2012). Finally, it is important to note that a study of interactions on these microscopic scales requires sampling and preservation of biological material collected from the environment that is not so destructive or invasive as to eliminate motility.

## Conclusion

Overall, the increasing recognition of the importance of the microbiome as a community of interacting microorganisms undoubtedly owes a great deal to analysis of metagenomes, metatranscriptomes and metaproteomes. As well as establishing the identity of the many species present in an environmental sample, these methods offer the means to determine the overall metabolic capability of a community. The increasing depth of coverage is now facilitating integrated systems approaches that can indicate hitherto unknown interactions. However, just as interactions between microbes are intricate, complex, and dynamic, an understanding of microbial ecology requires a combination of several interdisciplinary and complementary approaches. Defined combinations of known and well‐characterised partners in co‐culture in the laboratory have provided insight into the interactions at the molecular and cellular levels, the results of which often explain field observations, as well as indicate potential associations that are then validated in environmental samples. More importantly they are providing the basis to develop principles in ecology that represent the lifestyle and dynamics of microbial communities.

## Authorship

All authors contributed to the ideas in this manuscript and to its writing.
